# Central Nervous System Complications in COVID-19 Patients; a Systematic Review and Meta-Analysis based on Current Evidence

**Published:** 2020-06-07

**Authors:** Arian MadaniNeishaboori, Donya Moshrefiaraghi, Kosar Mohamed Ali, Amirmohammad Toloui, Mahmoud Yousefifard, Mostafa Hosseini

**Affiliations:** 1 Physiology Research Center, Iran University of Medical Sciences, Tehran, Iran.; 2 College of Medicine, University of Sulaimani, Sulaimani, Iraq.; 3 Department of Epidemiology and Biostatistics, School of Public Health, Tehran University of Medical Sciences, Tehran, Iran.

**Keywords:** COVID-19, Stroke, Prevalence, Central Nervous System Diseases

## Abstract

**Introduction:**

Several studies have confirmed neurological involvements, such as acute cerebrovascular disease and impaired consciousness in COVID-19. In the present study, our aim is to investigate the current evidence regarding central nervous system (CNS) complications in patients with confirmed COVID-19.

**Methods:**

An extensive search was conducted in electronic databases including Medline (using PubMed), Embase, Scopus, and Web of Science, in addition to the manual search in Google and Google scholar search engines, for articles published from 2019 until April 21st, 2020. Inclusion criteria was articles that were reviewed and reported the incidence of neurological outcomes in patients with confirmed COVID-19 disease.

**Results:**

The initial search yielded 638 records, from which 7 articles were included. Overall, the incidence of CNS complications was calculated to be 6.27% (95% CI: 3.32 to 9.98). The incidence of the most common CNS complications, encephalopathy and acute cerebrovascular disease, were 9.14% (95%CI: 2.20 to 19.81) and 2.59% (95% CI: 1.31 to 4.25), respectively.

**Conclusion:**

CNS complications do exist in COVID-19 patients, encephalopathy being the most concerning one. The heterogeneity in the existing literature causes an uncertainty in reporting the definite prevalence rate for each complication. Thus, further studies are needed for scientists to reach a more accurate estimate of the prevalence of these complications in COVID-19 patients. However, healthcare providers should consider the possibility of CNS involvements in patients with SARS-CoV-2 infection.

## Introduction

In December 2019, several cases of a severe inexplicable pneumonia emerged in Wuhan, China, and all those affected had connections to a local seafood market (1, 2). The etiology of the disease was confirmed to be a novel coronavirus (3). Due to the virus’s abysmal similarity to severe acute respiratory syndrome coronavirus (SARS-CoV) in clinical and genotype characteristics, it was named “SARS-CoV-2” or “severe acute respiratory syndrome coronavirus 2” by World Health Organization (WHO) on February 11^th^, 2020. At first, an environmental exposure was estimated to be the cause of the disease, but shortly after, with the number of patients rapidly increasing, human to human transmission was confirmed (4-6). After the disease spread to over 110 countries, WHO declared a global pandemic on March 11^th^ 2020, and as of that date, the number of COVID-19 cases is increasing daily (7).

The most common symptoms of the disease are fever, dry cough and dyspnea, in addition to fatigue, sore throat and other non-specific symptoms (6). Mostly, patients with diabetes, hypertension, cardiovascular diseases and old age suffer from severe type of the disease, and fatality rate is highest amongst these groups of patients (8, 9). Moreover, due to the virus’ host cell receptor being abundant in the body, the variety of its symptoms may expand even further. 

SARS-CoV-2 exploits the angiotensin converting enzyme 2 (ACE-2) receptor, expressed in many organs such as lungs, kidneys and neurological tissue, to enter the cells (10, 11). Due to that fact, the presence of ACE-2 receptor in neurological tissue may be the reason for SARS-CoV-2 having the potential to cause central nervous system (CNS) symptoms (12). In a retrospective case series conducted by Ling Mao et al., of 214 hospitalized COVID-19 patients in Wuhan, the epicenter of disease, 78 patients (36.4%) had developed neurological manifestations. These manifestations included acute cerebrovascular disease and impaired consciousness (13). Detailed neurological investigations such as autopsies and attempts to extract SARS-CoV-2 from cerebrospinal fluid and glial cells in COVID-19 patients, have indicated that cerebral involvement alone, can be a reason for the mortality caused by this disease ,due to the potential of causing cerebral edema (14). Overall, evidence confirming the presence of neurological involvement in COVID-19 do exist, so studies should be focused on illuminating the extent of that association.

Despite the fact that neurological complications particularly occur in severe forms of the disease (15-17), managing these complications can be vital to overall health and recovery of all COVID-19 patients. In this study, our aim is to review the current epidemiological evidence regarding CNS complications in patients whose COVID-19 infection has been confirmed by definitive laboratory test results.

## Methods

***Study design and setting***

The present systematic review and meta-analysis aims to acquire and analyze evidence regarding CNS outcomes in patients with COVID-19 disease. PICO in the present study is as follows: P: Patients with confirmed COVID-19, I: Report of a neurological outcome, O: Prevalence of the reported neurological outcome. Due to the purpose of this study, which was only reporting of neurological outcomes in patients with COVID-19 disease, no comparison was intended and thus, C in PICO was not defined. 

***Search strategy***

Firstly, several keywords were selected with the advice of experts in the field. Afterwards, selected keywords were searched in MeSH and Emtree to find related synonyms. Additionally, titles and abstracts of related articles were screened to find other possible relevant keywords. Finally, using the keywords, an extensive search was conducted in electronic databases Medline (using PubMed), Embase, Scopus and Web of Science, for articles published from 2019 until April 21^st^, 2020. Search strategy in Medline database through PubMed is presented in Appendix 1. In addition to the systematic search, manual search was also performed in Google and Google scholar to find additional, pre-printed manuscripts and possible missing articles.

***Selection criteria***

All articles that reviewed and reported the incidence of neurological outcomes in patients with confirmed COVID-19 were included in this review. Moreover, the exclusion criteria were case report articles, review articles and studies that only addressed neurological symptoms that cannot be certainly attributed to the involvement of CNS (such as general headache) in a COVID-19 patient. 

***Data collection***

Two independent researchers screened titles and abstracts of the articles obtained from searching the databases and gathered full texts of the possibly related studies. Next, based on the inclusion and exclusion criteria, articles were chosen and entered to the present systematic review and meta-analysis. Afterwards, the useful data of included articles were summarized and recorded. The recorded data included first author’s name, publication year, country in which the study was conducted in, number of patients, study design, number of patients in which the neurological outcome was assessed, mean age of the patients, number of males among the patients, type of reported neurological outcomes, diagnostic method used for recording the outcome, and number of patients presenting with the neurological outcome. Since some studies reported more than one neurological outcome, the prevalence rate was evaluated separately for each neurological outcome among the studies, as well as evaluating an overall rate of all neurological outcomes reported in the included studies. Any disagreements within the mentioned steps were resolved through discussion with a third reviewer.

***Quality assessment***

Since the design of included studies was observational, National Heart, Lung, and Blood Institute (NHLBI) quality assessment tool was used to evaluate the risk of bias among studies (18). Two independent reviewers assessed the studies and rated the items of the tool based on its key questions and personal judgment.

***Statistical analysis***

Analysis was performed in STATA 14.0 statistical program. Data was recorded as total sample size and frequency of CNS complication, and using “metaprop_one” command an overall prevalence with a 95% confidence interval (95% CI) was reported. Since, various CNS complications were reported in the included studies, we categorized them as acute cerebrovascular disease, brain encephalopathy and other complications (brain leptomeningeal enhancement, dysexecutive syndrome, brain perfusion abnormalities and ataxia) and analyses were stratified accordingly. I^2^ test was used to assess heterogeneity. Since considerable heterogeneity was observed, random effect model was adopted to estimate CNS complication and its corresponding 95% CI. Publication bias was assessed using Egger’s test.

## Results

***Study characteristics***

**Figure 1:**
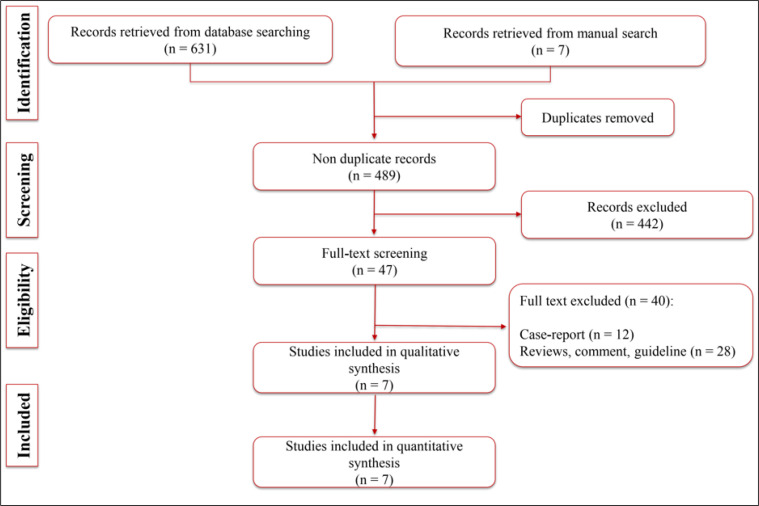
Flow diagram of the present meta-analysis

The initial search yielded 638 records, and after eliminating duplicates, 489 records remained. After reviewing the remaining studies, 7 articles were included in the present systematic review and meta-analysis (Figure 1) (13, 19-24). 4 studies were conducted in China, and the rest were conducted in France, Netherlands, and Italy. Only two Cohort studies were found among the included studies (21, 23), and the rest of the studies were either cross-sectional (20, 22, 24) or case series (1,7). In total, the included studies evaluated neurological outcomes in 1643 COVID-19 patients. The mean age of the studies patients ranged from 44 to 66 years old. The number of male patients was not recorded in one study (20); however, among the rest of the included studies, 974 males were studies among the 1585 recruited patients. Based on the included studies, encephalopathy was the most common CNS complication observed in 3 studies and among 45 COVID-19 patients (19, 20, 24), being evaluated based on clinical symptoms. Moreover, acute cerebrovascular disease and ischemic stroke was the second most common reported complication in the studies, being reported in 6 studies and 37 patients in total (13, 20-24). However, different studies used various diagnostic techniques to verify the incidence of acute cerebrovascular disease in patients, with computed tomography scan being the most common diagnostic method used. The other reported neurological complications observed following SARS-CoV-2 infection were brain leptomeningeal enhancement, dysexecutive syndrome, brain perfusion abnormalities (20) and ataxia (13). Table 1 summarizes characteristics of the 7 studies included in the present analysis. 

***Risk of bias assessment and publication bias***

**Figure 2:**
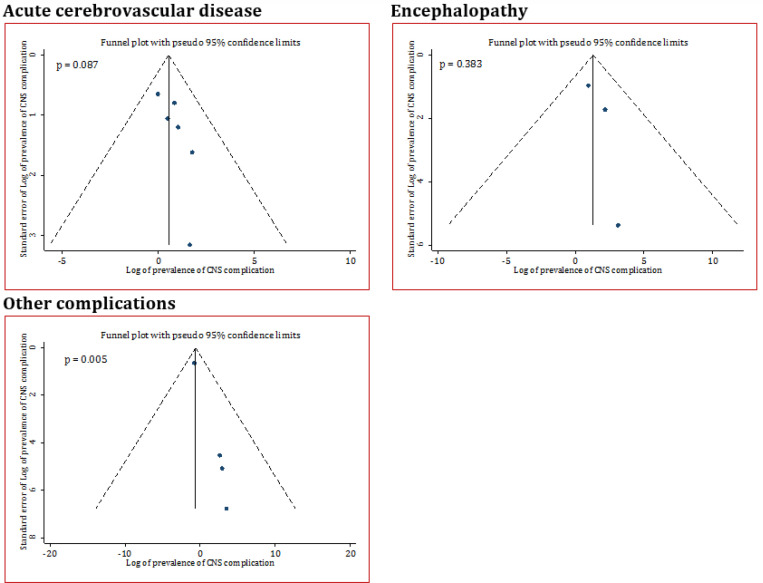
Funnel plot for assessment of publication bias in central nervous system (CNS) complications following SARS-CoV2 infection. Other complications: Brain leptomeningeal enhancement, dysexecutive syndrome, brain perfusion abnormalities, ataxia

No publication bias was observed in the studies reporting CNS outcomes based on Egger’s test and funnel plot assessment, as presented in Figure 2 (Acute cerebrovascular disease: P=0.087, Encephalopathy: p=0.383, other CNS complications: p=0.005). Risk of bias assessment of included studies is presented in table 2. Sample size justification was not provided in all studies. In addition, timeframe and follow up duration of 5 studies were not sufficient to evaluate association between COVID-19 and CNS manifestations, as some patients were still hospitalized at the time of analysis. Also, the blinding status of outcome assessor was not reported in some the studies. 

***Meta-analysis***

**Figure 3:**
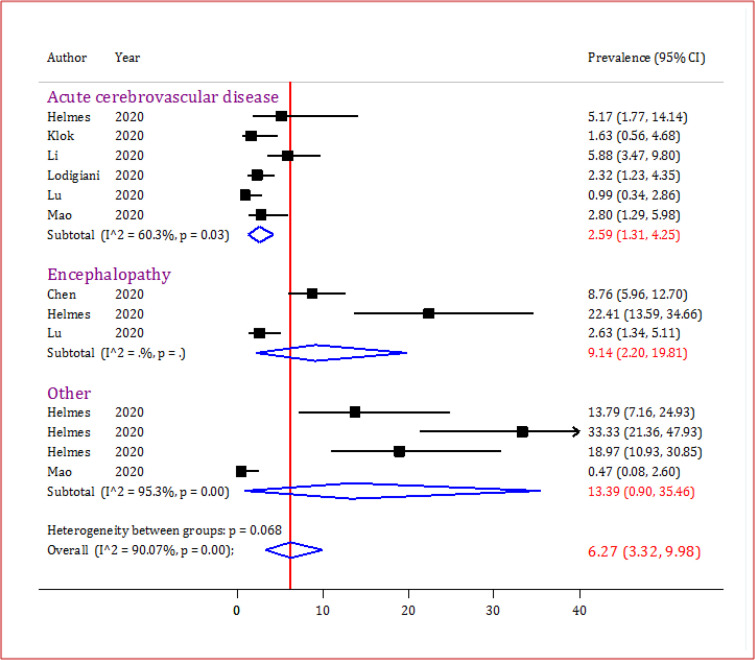
Forest plot for prevalence of central nervous system (CNS) complication following SARS-CoV-2 infection. The overall prevalence of CNS complication was 6.27%. Other complications: Brain leptomeningeal enhancement; Dysexecutive syndrome; Brain perfusion abnormalities; Ataxia

CNS complications were investigated and analyzed in the included studies, and results of the analysis are presented as a forest plot in Figure 3. Overall, the incidence of the CNS complications was calculated to be 6.27% (95% CI: 3.32 to 9.98) with a considerable heterogeneity observed between the studies as a whole (I^2^=90.07%). The prevalence of the acute cerebrovascular disease was 2.59% among COVID-19 patients (95% CI: 1.31 to 4.25; I^2^=60.3%). Moreover, overall reported incidence of encephalopathy was calculated to be 9.14% (95%CI: 2.20 to 19.81). Other CNS complications including brain leptomeningeal enhancement, dysexecutive syndrome, brain perfusion abnormalities and ataxia were also assessed and their overall incidence was evaluated to be 13.39% (95% CI 0.90 to 35.46; I^2^=95.3%).

**Table 1:** Summary of included studies

**Author; Year; Country**	**Study design**	**Sample size**	**Mean age**	**Number of males**	**Diagnostic methods**	**Type of neurologic manifestation**	**No. of complications**
Chen; 2020; China	Retrospective	274	62	171	Clinical symptom and laboratory findings	Hypoxic encephalopathy	24
Helmes; 2020; France	Prospective	58	63	NR	MRI	Cerebral ischemic stroke	3
					MRI	Brain leptomeningeal enhancement	8
					Clinical symptom	Encephalopathy	13
					Clinical symptom	Dysexecutive syndrome	15
					MRI	Brain perfusion abnormalities	11
Klok; 2020; Netherlands	Prospective	184	64	139	CT	Acute Cerebrovascular disease	3
Li; 2020; China	Retrospective	221	53.3	131	CT	Acute Cerebrovascular disease	13
Lodigiani; 2020; Italy	Retrospective	388	66	264	Report of treating physician	Acute Cerebrovascular disease	9
Lu; 2020; China	Retrospective	304	44	182	Clinical symptom	Encephalopathy	8
					NR	Acute cerebrovascular disease	3
Mao; 2020; China	Retrospective	214	52.7	87	CT	Acute cerebrovascular disease	6
					NR	Ataxia	1

CT: Computed tomography scan; MRI: Magnetic resonance imaging; NR: Not reported

**Table 2:** Risk of bias assessment of included studies based on NHLBI tools

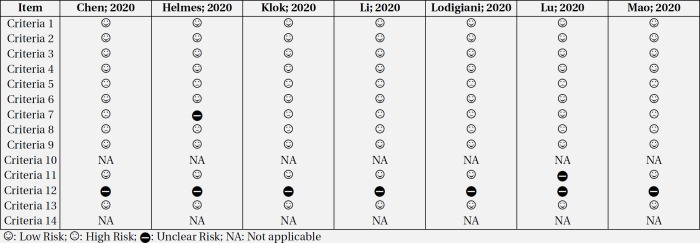

NA: Not applicable;

Criteria 1. Was the research question or objective in this paper clearly stated?

Criteria 2. Was the study population clearly specified and defined?

Criteria 3. Was the participation rate of eligible persons at least 50%?

Criteria 4. Were all the subjects selected or recruited from the same or similar populations (including the same time period)? Were inclusion and exclusion criteria for being in the study prespecified and applied uniformly to all participants?

Criteria 5. Was a sample size justification, power description, or variance and effect estimates provided?

Criteria 6. For the analyses in this paper, were the exposure(s) of interest measured prior to the outcome(s) being measured?

Criteria 7. Was the timeframe sufficient so that one could reasonably expect to see an association between exposure and outcome if it existed?

Criteria 8. For exposures that can vary in amount or level, did the study examine different levels of the exposure as related to the outcome (e.g., categories of exposure, or exposure measured as continuous variable)?

Criteria 9. Were the exposure measures (independent variables) clearly defined, valid, reliable, and implemented consistently across all study participants?

Criteria 10. Was the exposure(s) assessed more than once over time?

Criteria 11. Were the outcome measures (dependent variables) clearly defined, valid, reliable, and implemented consistently across all study participants?

Criteria 12. Were the outcome assessors blinded to the exposure status of participants?

Criteria 13. Was loss to follow-up after baseline 20% or less?

Criteria 14. Were key potential confounding variables measured and adjusted statistically for their impact on the relationship between exposure(s) and outcome(s)?

**Appendix 1:** Medline search query

Search terms
“Betacoronavirus”[mh] OR “Coronavirus”[mh] OR “Coronavirus Infections”[mh] OR "COVID-19 vaccine"[Supplementary Concept] OR "COVID-19 diagnostic testing"[Supplementary Concept] OR 2019 novel coronavirus disease[tiab] OR COVID19[tiab] OR COVID-19[tiab] OR SARS-CoV-2[tiab] OR COVID-19 pandemic[tiab] OR SARS-CoV-2 infection[tiab] OR COVID-19 virus disease[tiab] OR 2019 novel coronavirus infection[tiab] OR 2019-nCoV infection[tiab] OR coronavirus disease 2019[tiab] OR coronavirus disease-19[tiab] OR 2019-nCoV disease[tiab] OR COVID-19 virus infection[tiab] OR Severe Acute Respiratory Syndrome Coronavirus 2[tiab] “Stroke”[mh] OR “Cerebrovascular Disorders”[mh] OR “Brain Ischemia”[mh] OR “Encephalitis”[mh] OR “Infectious Encephalitis”[mh] OR “Encephalitis, Viral”[mh] OR “Encephalitis Viruses”[mh] OR “Meningitis”[mh] OR “Meningitis, Viral”[mh] OR “Central Nervous System Infections”[mh] OR “Central Nervous System Viral Diseases”[mh] OR “Nervous System Diseases”[mh] OR “Central Nervous System Diseases”[mh] OR “Neurologic Manifestations”[mh] OR “Brain”[mh] OR “Brain Infarction”[mh] OR Stroke[tiab] OR Strokes[tiab] OR Cerebrovascular Accident[tiab] OR Cerebrovascular Accidents[tiab] OR CVA (Cerebrovascular Accident)[tiab] OR CVAs (Cerebrovascular Accident)[tiab] OR Cerebrovascular Apoplexy[tiab] OR Apoplexy, Cerebrovascular[tiab] OR Vascular Accident, Brain[tiab] OR Brain Vascular Accident[tiab] OR Brain Vascular Accidents[tiab] OR Vascular Accidents, Brain[tiab] OR Cerebrovascular Stroke[tiab] OR Cerebrovascular Strokes[tiab] OR Stroke, Cerebrovascular[tiab] OR Strokes, Cerebrovascular[tiab] OR Apoplexy[tiab] OR Cerebral Stroke[tiab] OR Cerebral Strokes[tiab] OR Stroke, Cerebral[tiab] OR Strokes, Cerebral[tiab] OR Stroke, Acute[tiab] OR Acute Stroke[tiab] OR Acute Strokes[tiab] OR Strokes, Acute[tiab] OR Cerebrovascular Accident, Acute[tiab] OR Acute Cerebrovascular Accident[tiab] OR Acute Cerebrovascular Accidents[tiab] OR Cerebrovascular Accidents, Acute[tiab] OR Brain Infarction[tiab] OR Brain Infarctions[tiab] OR Infarction, Brain[tiab] OR Infarctions, Brain[tiab] OR Brain Infarct[tiab] OR Brain Infarcts[tiab] OR Infarct, Brain[tiab] OR Infarcts, Brain[tiab] OR Anterior Circulation Brain Infarction[tiab] OR Infarction, Brain, Anterior Circulation[tiab] OR Infarction, Anterior Circulation, Brain[tiab] OR Anterior Circulation Infarction, Brain[tiab] OR Brain Infarction, Anterior Circulation[tiab] OR Venous Infarction, Brain[tiab] OR Brain Venous Infarction[tiab] OR Brain Venous Infarctions[tiab] OR Infarction, Brain Venous[tiab] OR Infarctions, Brain Venous[tiab] OR Venous Infarctions, Brain[tiab] OR Brain Infarction, Venous[tiab] OR Brain Infarctions, Venous[tiab] OR Infarction, Venous Brain[tiab] OR Infarctions, Venous Brain[tiab] OR Venous Brain Infarction[tiab] OR Venous Brain Infarctions[tiab] OR Anterior Cerebral Circulation Infarction[tiab] OR Infarction, Anterior Cerebral Circulation[tiab] OR Brain Infarction, Posterior Circulation[tiab] OR Posterior Circulation Infarction, Brain[tiab] OR Posterior Circulation Brain Infarction[tiab] OR Infarction, Brain, Posterior Circulation[tiab] OR Infarction, Posterior Circulation, Brain[tiab] OR Cerebrovascular Disorders[tiab] OR Cerebrovascular Disorder[tiab] OR Vascular Diseases, Intracranial[tiab] OR Intracranial Vascular Disease[tiab] OR Intracranial Vascular Diseases[tiab] OR Vascular Disease, Intracranial[tiab] OR Intracranial Vascular Disorders[tiab] OR Intracranial Vascular Disorder[tiab] OR Vascular Disorder, Intracranial[tiab] OR Vascular Disorders, Intracranial[tiab] OR Cerebrovascular Diseases[tiab] OR Cerebrovascular Disease[tiab] OR Disease, Cerebrovascular[tiab] OR Diseases, Cerebrovascular[tiab] OR Brain Vascular Disorders[tiab] OR Brain Vascular Disorder[tiab] OR Vascular Disorder, Brain[tiab] OR Vascular Disorders, Brain[tiab] OR Cerebrovascular Occlusion[tiab] OR Cerebrovascular Occlusions[tiab] OR Occlusion, Cerebrovascular[tiab] OR Occlusions, Cerebrovascular[tiab] OR Cerebrovascular Insufficiency[tiab] OR Cerebrovascular Insufficiencies[tiab] OR Insufficiencies, Cerebrovascular[tiab] OR Insufficiency, Cerebrovascular[tiab] OR Brain Ischemia[tiab] OR Brain Ischemias[tiab] OR Ischemia, Brain[tiab] OR Ischemic Encephalopathy[tiab] OR Encephalopathy, Ischemic[tiab] OR Ischemic Encephalopathies[tiab] OR Cerebral Ischemia[tiab] OR Cerebral Ischemias[tiab] OR Ischemias, Cerebral[tiab] OR Ischemia, Cerebral[tiab] OR Encephalitis[tiab] OR Brain Inflammation[tiab] OR Inflammation, Brain[tiab] OR Brain Inflammations[tiab] OR Rasmussen Syndrome[tiab] OR Rasmussen Encephalitis[tiab] OR Rasmussen's Syndrome[tiab] OR Encephalitis, Rasmussen[tiab] OR Infectious Encephalitis[tiab] OR Encephalitis, Infectious[tiab] OR Encephalitis Infection[tiab] OR Encephalitis Infections[tiab] OR Infection, Encephalitis[tiab] OR Infections, Encephalitis[tiab] OR Encephalitis, Viral[tiab] OR Encephalomyelitis, Infectious, Viral[tiab] OR Infectious Encephalomyelitis, Viral[tiab] OR Encephalomyelitis, Viral Infectious[tiab] OR Viral Infectious Encephalomyelitis[tiab] OR Viral Encephalitis[tiab] OR Encephalitis Viruses[tiab] OR Viruses, Encephalitis[tiab] OR Encephalitis Virus[tiab] OR Virus, Encephalitis[tiab] OR Meningitis[tiab] OR Meningitides[tiab] OR Pachymeningitis[tiab] OR Pachymeningitides[tiab] OR Meningitis, Viral[tiab] OR Meningitides, Viral[tiab] OR Viral Meningitides[tiab] OR Viral Meningitis[tiab] OR Central Nervous System Infections[tiab] OR Infections, Central Nervous System[tiab] OR CNS Infections[tiab] OR CNS Infection[tiab] OR Infection, CNS[tiab] OR Infections, CNS[tiab] OR Central Nervous System Infection[tiab] OR Central Nervous System Viral Diseases[tiab] OR Viral Diseases, Central Nervous System[tiab] OR Viral Infections, Central Nervous System[tiab] OR Infections, CNS, Viral[tiab] OR Infections, Viral CNS[tiab] OR CNS Infection, Viral[tiab] OR CNS Infections, Viral[tiab] OR Infection, Viral CNS[tiab] OR Viral CNS Infection[tiab] OR Viral CNS Infections[tiab] OR CNS Viral Diseases[tiab] OR CNS Viral Disease[tiab] OR Disease, CNS Viral[tiab] OR Diseases, CNS Viral[tiab] OR Viral Disease, CNS[tiab] OR Viral Diseases, CNS[tiab] OR Central Nervous System Viral Infections[tiab] OR Nervous System Diseases[tiab] OR Disease, Nervous System[tiab] OR Diseases, Nervous System[tiab] OR Nervous System Disease[tiab] OR Neurologic Disorders[tiab] OR Disorder, Neurologic[tiab] OR Disorders, Neurologic[tiab] OR Neurologic Disorder[tiab] OR Neurological Disorders[tiab] OR Disorder, Neurological[tiab] OR Disorders, Neurological[tiab] OR Neurological Disorder[tiab] OR Nervous System Disorders[tiab] OR Disorder, Nervous System[tiab] OR Disorders, Nervous System[tiab] OR Nervous System Disorder[tiab] OR Central Nervous System Diseases[tiab] OR Central Nervous System Disorders[tiab] OR CNS Diseases[tiab] OR CNS Disease[tiab] OR Neurologic Manifestations[tiab] OR Manifestation, Neurologic[tiab] OR Neurological Manifestations[tiab] OR Neurologic Manifestation[tiab] OR Neurologic Signs and Symptoms[tiab] OR Manifestations, Neurologic[tiab] OR Manifestations, Neurological[tiab] OR Manifestation, Neurological[tiab] OR Neurological Manifestation[tiab] OR Neurologic Deficits[tiab] OR Deficit, Neurologic[tiab] OR Deficits, Neurologic[tiab] OR Neurologic Deficit[tiab] OR Neurologic Symptoms[tiab] OR Neurologic Symptom[tiab] OR Symptom, Neurologic[tiab] OR Symptoms, Neurologic[tiab] OR Neurologic Findings[tiab] OR Finding, Neurologic[tiab] OR Findings, Neurologic[tiab] OR Neurologic Finding[tiab] OR Neurologic Signs[tiab] OR Neurologic Sign[tiab] OR Sign, Neurologic[tiab] OR Signs, Neurologic[tiab] OR Focal Neurologic Deficits[tiab] OR Deficit, Focal Neurologic[tiab] OR Deficits, Focal Neurologic[tiab] OR Focal Neurologic Deficit[tiab] OR Neurologic Deficit, Focal[tiab] OR Neurologic Deficits, Focal[tiab] OR Neurologic Dysfunction[tiab] OR Dysfunction, Neurologic[tiab] OR Dysfunctions, Neurologic[tiab] OR Neurologic Dysfunctions[tiab] OR Encephalopathy[tiab] OR Clinical characteristics[tiab] OR Brain[tiab] #1 AND #2

## Discussion

The findings of the present systematic review and meta-analysis demonstrated that CNS complications definitely exist in COVID-19 patients, with an overall prevalence calculated to be 6.27% based on the studies found until April 21^st^ 2020. In terms of the type of complications, encephalopathy and acute cerebrovascular disease were the most prevalent, and other CNS complications including brain leptomeningeal enhancement, dysexecutive syndrome, brain perfusion abnormalities and ataxia were also observed among the patients. However, there are a few limitations regarding the reported prevalence.

Regarding the incidence of encephalopathy, although it was the most prevalent CNS complication with respect to the overall number of patients, only three articles reported this complication (19, 20, 24). Moreover, the study sample size in the three articles differ, with Helmes et al. contemplating on a patient population much less than the other two studies, causing a possible bias in the results of the study. Also, Helmes et al. studied only a population of severe cases of COVID-19 patients, whilst the other two studies contemplated on a spectrum of disease severities among their included study samples. As a result, more studies with larger population and variety in severity of disease among patients are needed to reach a more accurate consensus over the actual prevalence of encephalopathy between COVID-19 patients. Nonetheless, the possibility of encephalopathy is noticeably high, and thus, healthcare providers should pay extra attention to its presence in patients with COVID-19. Previously, some case report studies had reported the incidence of encephalopathy as a complication of COVID-19 (25-27), and our results have raised more concern over this matter.

 Six studies reported the incidence of acute cerebrovascular disease and stroke in COVID-19 patients. However, two studies in this section may be the source of existing heterogeneity (20, 22), reporting a higher than average prevalence for acute cerebrovascular disease and stroke. As previously mentioned, the sample size in Helmes et al.’s study is considerably low compared with the other five articles. In addition, concerning other neurological complications, only two studies recorded and reported neurological complications other than acute cerebrovascular disease and encephalopathy (13, 20), so more researches are required to take place to evaluate and assess other types of CNS complications in COVID-19 patients.

## Conclusion:

Overall, we conclude that CNS complications in COVID-19 patients do exist. However, with the studies reporting a variety of types of complications, and the focus of the existing studies being mainly on encephalopathy and acute cerebrovascular disease, there is inevitably uncertainty. Nonetheless, our results have emphasized that SARS-CoV-2 may damage the CNS while infecting its host (28, 29). In this regard, healthcare providers should take extra care with their COVID-19 patients presenting with symptoms indicative of CNS complications.

## References

[1] Wang C, Horby PW, Hayden FG, Gao GF (2020). A novel coronavirus outbreak of global health concern. The Lancet.

[2] Huang C, Wang Y, Li X, Ren L, Zhao J, Hu Y (2020). Clinical features of patients infected with 2019 novel coronavirus in Wuhan, China. The lancet.

[3] Zhu N, Zhang D, Wang W, Li X, Yang B, Song J (2020). China Novel Coronavirus Investigating and Research Team A novel coronavirus from patients with pneumonia in China, 2019. N Engl J Med.

[4] Chan JF-W, Yuan S, Kok K-H, To KK-W, Chu H, Yang J (2020). A familial cluster of pneumonia associated with the 2019 novel coronavirus indicating person-to-person transmission: a study of a family cluster. The Lancet.

[5] Wang D, Hu B, Hu C, Zhu F, Liu X, Zhang J (2020). Clinical characteristics of 138 hospitalized patients with 2019 novel coronavirus–infected pneumonia in Wuhan, China. Jama.

[6] Guan W-j, Ni Z-y, Hu Y, Liang W-h, Ou C-q, He J-x (2020). Clinical characteristics of coronavirus disease 2019 in China. New England journal of medicine.

[7] Organization WH (2020). Coronavirus disease 2019 (‎ COVID-19)‎: situation report, 92.

[8] Guan W-j, Liang W-h, Zhao Y, Liang H-r, Chen Z-s, Li Y-m (2020). Comorbidity and its impact on 1590 patients with Covid-19 in China: A Nationwide Analysis. European Respiratory Journal.

[9] Zhou F, Yu T, Du R, Fan G, Liu Y, Liu Z (2020). Clinical course and risk factors for mortality of adult inpatients with COVID-19 in Wuhan, China: a retrospective cohort study. The lancet.

[10] Netland J, Meyerholz DK, Moore S, Cassell M, Perlman S (2008). Severe acute respiratory syndrome coronavirus infection causes neuronal death in the absence of encephalitis in mice transgenic for human ACE2. Journal of virology.

[11] Lu R, Zhao X, Li J, Niu P, Yang B, Wu H (2020). Genomic characterisation and epidemiology of 2019 novel coronavirus: implications for virus origins and receptor binding. The Lancet.

[12] Wan Y, Shang J, Graham R, Baric RS, Li F (2020). Receptor recognition by the novel coronavirus from Wuhan: an analysis based on decade-long structural studies of SARS coronavirus. Journal of virology.

[13] Mao L, Jin H, Wang M, Hu Y, Chen S, He Q (2020). Neurologic manifestations of hospitalized patients with coronavirus disease 2019 in Wuhan, China. JAMA neurology.

[14] Baig AM, Khaleeq A, Ali U, Syeda H (2020). Evidence of the COVID-19 virus targeting the CNS: tissue distribution, host–virus interaction, and proposed neurotropic mechanisms. ACS chemical neuroscience.

[15] He X, Lai J, Cheng J, Wang M, Liu Y, Xiao Z (2020). Impact of complicated myocardial injury on the clinical outcome of severe or critically ill COVID-19 patients. Zhonghua xin xue guan bing za zhi.

[16] Metlay JP, Waterer GW, Long AC, Anzueto A, Brozek J, Crothers K (2019). Diagnosis and treatment of adults with community-acquired pneumonia An official clinical practice guideline of the American Thoracic Society and Infectious Diseases Society of America. American journal of respiratory and critical care medicine.

[17] Li Y, Li H, Fan R, Wen B, Zhang J, Cao X (2016). Coronavirus infections in the central nervous system and respiratory tract show distinct features in hospitalized children. Intervirology.

[18] Health NIo (2014). Study Quality Assessment Tools| National Heart, Lung, and Blood Institute (NHLBI). National Institutes of Health.

[19] Chen T, Wu D, Chen H, Yan W, Yang D, Chen G (2020). Clinical characteristics of 113 deceased patients with coronavirus disease 2019: retrospective study. Bmj.

[20] Helms J, Kremer S, Merdji H, Clere-Jehl R, Schenck M, Kummerlen C (2020). Neurologic features in severe SARS-CoV-2 infection. New England Journal of Medicine.

[21] Klok FA, Kruip MJHA, van der Meer NJM, Arbous MS, Gommers DAMPJ, Kant KM (2020). Incidence of thrombotic complications in critically ill ICU patients with COVID-19. Thrombosis Research.

[22] Li Y, Wang M, Zhou Y, Chang J, Xian Y, Mao L (2020). Acute cerebrovascular disease following COVID-19: a single center, retrospective observational study.

[23] Lodigiani C, Iapichino G, Carenzo L, Cecconi M, Ferrazzi P, Sebastian T (2020). Venous and arterial thromboembolic complications in COVID-19 patients admitted to an academic hospital in Milan, Italy. Thrombosis research.

[24] Lu L, Xiong W, Liu D, Liu J, Yang D, Li N (2020). New‐onset acute symptomatic seizure and risk factors in Corona Virus Disease 2019: A Retrospective Multicenter Study. Epilepsia.

[25] Filatov A, Sharma P, Hindi F, Espinosa PS (2020). Neurological complications of coronavirus disease (COVID-19): encephalopathy. Cureus.

[26] Poyiadji N, Shahin G, Noujaim D, Stone M, Patel S, Griffith B COVID-19–associated acute hemorrhagic necrotizing encephalopathy: CT and MRI features. Radiology.

[27] Moriguchi T, Harii N, Goto J, Harada D, Sugawara H, Takamino J (2020). A first Case of Meningitis/Encephalitis associated with SARS-Coronavirus-2. International Journal of Infectious Diseases.

[28] Turtle L (2020). Respiratory failure alone does not suggest central nervous system invasion by SARS‐CoV‐2. Journal of Medical Virology.

[29] Wu Y, Xu X, Chen Z, Duan J, Hashimoto K, Yang L (2020). Nervous system involvement after infection with COVID-19 and other coronaviruses. Brain, Behavior, and Immunity.

